# T3SS1 accessory protein gene *vscW* alters motility, biofilm formation, outer membrane homeostasis, and virulence in *Vibrio parahaemolyticus*

**DOI:** 10.3389/fcimb.2026.1961181

**Published:** 2026-09-25

**Authors:** Yuanhao Zhang, Chunyuan Wang, Yingeng Wang, Zheng Zhang, Yong-An Zhang, Yongxiang Yu, Zhiqi Zhang, Xiaojun Rong, Xinzhang Ji

**Affiliations:** 1 State Key Laboratory of Mariculture Biobreeding and Sustainable Goods, Yellow Sea Fisheries Research Institute, Chinese Academy of Fishery Sciences, Qingdao, Shandong, China; 2 College of Fisheries, Huazhong Agricultural University, Wuhan, Hubei, China; 3 Laboratory of Marine Fishery Science and Food Yield Process, Qingdao Marine Science and Technology Center, Qingdao, Shandong, China; 4 Hebei Yuanzhang Aquaculture Co., Ltd., Cangzhou, Hebei, China

**Keywords:** biofilm formation, outer membrane homeostasis, pathogenicity, proteomics, type III secretion system 1, *Vibrio parahaemolyticus*, vscW

## Abstract

*Vibrio parahaemolyticus* is a Gram-negative pathogen widely distributed in marine environments and can cause infections in humans and aquatic animals. Type III secretion system 1 (T3SS1) accessory protein VscW is homologous to YscW from *Yersinia*, which contributes to the stability of the T3SS outer membrane secretin YscC and facilitates its oligomerization and localization to the outer membrane. However, the specific function of *vscW* in *V. parahaemolyticus* remains poorly understood. In this study, a *vscW* deletion mutant (Δ*vscW*-P2C) was constructed by homologous recombination. Compared with the wild-type strain (WT-P2C), Δ*vscW*-P2C exhibited 41.1% and 45.0% reductions in swimming motility and biofilm formation, respectively. N-phenyl-1-naphthylamine (NPN) fluorescence intensity increased by 60.7% in Δ*vscW*-P2C, indicating enhanced outer membrane permeability. Pathogenicity examination showed that the cumulative mortality of *Litopenaeus vannamei* decreased from 66.7% to 41.7% after deletion of the *vscW* gene, representing an approximate 37.5% reduction in mortality. Proteomic analysis identified 243 differentially expressed proteins, mainly involving T3SS-associated proteins (VscC and VopB), T6SS-associated proteins (Hcp, VgrG, VasG, ImpC, and ImpL), c-di-GMP-associated proteins (GGDEF and EAL-domain-containing proteins), biofilm-associated proteins (VpsT-like, GlnA, and GST), motility- and chemotaxis-associated proteins (FliC and MCP), and putative virulence-associated proteins (PAAR-like-AHH, TcpQ, and VesC). Together, these phenotypic and proteomic findings suggest that *vscW* deletion is associated with alterations in T3SS1 and other virulence-related functional networks. Collectively, our findings support a contribution of VscW to virulence-associated phenotypes and pathogenicity in *V. parahaemolyticus* and provide a basis for further investigation of the underlying regulatory mechanisms.

## Introduction

*V. parahaemolyticus* is a Gram-negative halophilic bacterium widely distributed in aquatic environments, including estuarine and marine ecosystems (Dubey et al., 2021; Brumfield et al., 2023; Bai et al., 2024b). It can infect various aquatic animals, including shrimp, crabs, oysters, and clams (Yeung and Boor, 2004; Su and Liu, 2007), and is an important foodborne pathogen responsible for seafood-associated acute gastroenteritis in humans (Stratev et al., 2021).

The pathogenicity of *V. parahaemolyticus* is collectively mediated by multiple virulence factors, including thermostable direct hemolysin (TDH), TDH-related hemolysin (TRH), thermolabile hemolysin (TLH), adhesins, type III secretion system (T3SS), and type VI secretion system (T6SS) (Broberg et al., 2011). Among these factors, T3SS is an important virulence determinant with a structure resembling a needle-like syringe. This apparatus enables bacteria to translocate effector proteins in an unfolded state into host cells, where they refold into their active conformations and disrupt host cell signaling, cytoskeletal homeostasis, and immune responses (Makino et al., 2003; Caron et al., 2006; Paranjpye et al., 2012). Two T3SSs, T3SS1 and T3SS2, have been identified in *V. parahaemolyticus* (Tagomori et al., 2002). The T3SS1 gene cluster is present in both non-pathogenic and pathogenic strains, whereas the T3SS2 gene cluster is primarily associated with pathogenic strains and enterotoxicity (Okada et al., 2010; Paranjpye et al., 2012).

T3SS1 is an important virulence apparatus that mediates the cytotoxicity of *V. parahaemolyticus* and can induce autophagy, apoptosis, or lysis in host cells (Hiyoshi et al., 2010). Within the T3SS1 gene cluster, *vscW* encodes VscW, an accessory protein belonging to the YscW family (Vanden Bergh and Frey, 2014); however, its specific function in *V. parahaemolyticus* remains unclear. T3SS1 consists of structural, regulatory, translocator, effector, and accessory proteins, and its expression is precisely controlled by regulatory factors such as ExsA, ExsC, ExsD, and ExsE (Ono et al., 2006; Zhou et al., 2008; Kodama et al., 2010). Previous studies have demonstrated that the translocon components VopB1 and VopD1 (Shimohata et al., 2012) and the T3SS1 needle protein VscF (Lian et al., 2021) participate in T3SS1-mediated effector translocation and virulence. In contrast, the functions of accessory proteins such as VscW remain poorly understood. These accessory proteins generally do not act directly on host cells but maintain efficient protein translocation by facilitating the localization, stabilization, and assembly of key structural components (Parsot et al., 2003; Korotkov et al., 2011; Büttner, 2012; Korotkov et al., 2012).

Current hypotheses regarding the function of *vscW* are primarily based on its homologue YscW, and other T3SS accessory proteins. In *Yersinia*, YscW promotes oligomerization and stabilization of the outer membrane secretin YscC and facilitates its correct localization in the outer membrane. Deletion of *yscW* reduces YscC oligomer formation and outer membrane localization and markedly impairs T3SS-mediated protein secretion (Burghout et al., 2004). In *Yersinia* Ysa T3SS, the pilotin protein YsaP similarly participates in the outer membrane localization of the secretin YsaC, and its deletion impairs T3SS-mediated protein export (Rau and Darwin, 2015). MxiM in *Shigella* and InvH in *Salmonella* perform similar functions by contributing to the outer membrane localization and stabilization of secretins MxiD and InvG, respectively, thereby affecting T3SS apparatus assembly and effector secretion (Daefler and Russel, 1998; Lario et al., 2005). These findings demonstrate that T3SS accessory proteins are closely associated with the assembly and function of the secretion apparatus and provide a basis for hypothesizing that *vscW* may contribute to the maintenance of T3SS1 function in *V. parahaemolyticus*. However, these findings were mainly obtained from *Yersinia* and other bacterial species, and therefore cannot directly establish the specific function of *vscW* in *V. parahaemolyticus*. Whether *vscW* deletion affects T3SS1-associated functions and whether such effects subsequently alter bacterial motility, biofilm formation, outer membrane homeostasis, and host pathogenicity remains unclear.

Accordingly, this study focused on *vscW* in the *V. parahaemolyticus* P2C strain. A *vscW* deletion mutant and complemented strain were constructed using suicide plasmid-mediated homologous recombination, and their biological characteristics were analyzed to investigate the role of *vscW* in the regulation of *V. parahaemolyticus virulence.*

## Materials and methods

### Bacterial strains and plasmids

The bacterial strains and plasmids used in this study are listed in **Table 1**. The wild-type strain WT-P2C, donor strain *Escherichia coli* S17-1 λpir, and plasmids pRE112 and pBBR1MCS-5 were stored in our laboratory for subsequent use. Healthy Pacific white shrimp (*Litopenaeus vannamei*) were obtained from a farm in Qingdao, Shandong Province, China.

**Table 1:** Bacterial strains and plasmids used in this study.

Strains and plasmids	Description	Source
WT-P2C	wild-type *V. parahaemolyticus* strain, Ampr	Laboratory stock
*ΔvscW*-P2C	*V. parahaemolyticus vscW* deletion mutant, Ampr	Constructed in this study
C*ΔvscW*-P2C	*V. parahaemolyticus vscW*-complemented strain, Ampr, Gmr	Constructed in this study
*E. coli* S17-1 λpir	Donor strain used for conjugative transfer	Laboratory stock
pRE112	Suicide plasmid, sacB, Cmr	Laboratory stock
pBBR1MCS-5	Broad-host-range shuttle plasmid, Gmr	Laboratory stock
pRE112-UD*vscW*	Used to construct the *vscW* deletion mutant	Constructed in this study
pBBR1-P+*vscW*	Used to construct the *vscW*-complemented strain	Constructed in this study

### Construction of *ΔvscW*-P2C and C*ΔvscW*-P2C

An allelic exchange method based on the suicide plasmid pRE112 was used to construct the *vscW* deletion mutant *ΔvscW*-P2C, as previously described (Matsuo et al., 2013). Genomic DNA was extracted from WT-P2C and used as a template to amplify the upstream and downstream homologous arms of *vscW* using the primer pairs *vscW*-1F/*vscW*-1R and *vscW*-2F/*vscW*-2R. The amplified upstream and downstream homologous arms were ligated to pRE112 using T4 DNA ligase to generate the recombinant plasmid pRE112-UD*vscW*. After verification by PCR and sequencing, the recombinant plasmid was introduced into the donor strain *E. coli* S17-1 λpir and confirmed by PCR using the primers pRE112-F/pRE112-R. The donor carrying the recombinant plasmid was then conjugated to the recipient WT-P2C. Positive clones resulting from the first crossover were selected on TCBS agar plates containing chloramphenicol and ampicillin (Amp). After PCR verification, the first-crossover strains were plated on TSB agar containing 20% sucrose to select candidate second-crossover strains for further analysis. Candidate strains were identified using PCR with internal primers. Following three to five serial passages and repeated PCR verification, a genetically stable *vscW* deletion mutant (*ΔvscW*-P2C) was generated.

A broad-host-range plasmid, pBBR1MCS-5, was used to generate *vscW*-complemented strains. WT-P2C genomic DNA was used as a template to amplify the *vscW*-containing complementation fragment using the primers *vscW*-HF and *vscW*-HR. The fragment was ligated into pBBR1MCS-5 and introduced into *E. coli* S17-1 λpir. Primers pBBR1-F and pBBR1-R were used to verify the successful introduction of the recombinant plasmids. A donor carrying a sequence-verified plasmid was conjugated with the Δ*vscW*-P2C mutant. Positive strains carrying the complementation plasmid were selected on TCBS agar containing gentamicin and ampicillin and confirmed using PCR. Positive single colonies were serially passaged three to five times until the construct was stably maintained, yielding a complemented strain. The primers used for strain construction are listed in **Table 2**.

**Table 2:** Primers used for construction of the *vscW* deletion mutant and complemented strain.

Primer name	Primer sequence (5′–3′)	Product size (bp)
*vscW*-1F	TCCGGCAATAACTGAAATCG	506
*vscW*-1R	AAGAGGAAGCGTGGGAC	
*vscW*-2F	GAACTGTCCGCACGAGA	493
*vscW*-2R	TCAGCCAGCAACGATAAGG	
pRE112-F	CGGGTTGAGAAGCGGTGTA	1194(pRE112-UD*vscW)*
pRE112-R	CAGCCAATCCCTGGGTGAG	
in-*vscW*-F	GCCAACATAAGGAAAAGT	240 (Δ*vscW*)
in-*vscW*-R	TAGCAACCAACACCAAG	
*vscW*-HF	TCGCCCAAGTCGTGTTC	1983
*vscW*-HR	TCAATCCAACTCATCGTCCC	
pBBR1-F	GTAACGCCAGGGTTTTCC	2153(pBBR1-P+*vscW*)
pBBR1-R	GGCTTTACACTTTATGCTTCC	

### Transmission electron microscopy

Following the method described by Bai et al. (2024a) with minor modifications, purified single colonies of WT-P2C and *ΔvscW*-P2C were separately inoculated onto TSB agar plates supplemented with 1.5% NaCl. The following day, colonies were gently collected from the agar surface and resuspended in sterile buffer. An appropriate volume of the bacterial suspension was placed on a clean glass slide, and a copper grid was floated on the surface of the suspension for 2 min. The excess suspension was then carefully removed. The grid was negatively stained with 5% phosphotungstic acid for 1 min, blotted to remove excess stain, washed once with ddH_2_O, and allowed to air dry. Finally, the cellular morphology and flagellar structures of WT-P2C and *ΔvscW*-P2C were examined using transmission electron microscopy.

### Growth curve assay

To assess the effect of *vscW* deletion on P2C growth, growth curves were determined for WT-P2C, Δ*vscW*-P2C, and CΔ*vscW*-P2C cells. The three strains were cultured in the same growth phase and the bacterial suspensions were adjusted to an OD_600_ of approximately 0.5. The suspensions were then inoculated into fresh sterile TSB at a 1:100 ratio and dispensed into 96-well plates at 200 μL per well, with three technical replicate wells for each strain tested. Sterile TSB was used as a blank control. The OD_600_ was measured every 2 h during incubation, and growth curves were plotted based on these measurements. The experiments were performed in triplicate.

### Motility assays

The WT-P2C and Δ*vscW*-P2C strains were activated on TSB agar supplemented with 1.5% sodium chloride (NaCl). Single colonies were picked with sterile toothpicks and stab-inoculated vertically into the centers of semi-solid TSB media containing 0.3% or 0.6% agar. The plates were then incubated at 28 °C. Colony morphology and expansion were visually observed at 12 h, whereas the colony expansion diameters were measured at 24 h for quantitative analysis. After incubation, the plates were imaged using a Scan 1200 automatic colony counter and inhibition zone reader (Interscience, France), and the migration-zone diameters were measured using the accompanying Scan Interscience software. Each colony expansion diameter was measured three times, and the entire experiment was independently repeated three times.

### Biofilm formation assay

To assess the effect of *vscW* deletion on P2C biofilm formation, we measured biofilm formation by WT-P2C, *ΔvscW*-P2C, and C*ΔvscW*-P2C using crystal violet staining. Suspensions of the three strains were adjusted to an OD_600_ of approximately 0.6, inoculated into fresh sterile TSB broth at a 1:100 ratio, and dispensed into sterile 96-well plates at 200 µL per well. Six technical replicate wells were prepared for each strain, with uninoculated TSB serving as a blank control. Unused wells were filled with equal volumes of sterile water to minimize evaporation during incubation. The plates were sealed and incubated at 28 °C for 48 h.

After incubation, the cultures were carefully aspirated, and the experimental wells were gently washed three times with phosphate-buffered saline (PBS) to remove non-adherent cells. After air-drying, each well was fixed with 200 μL of methanol for 15 min, the fixative was discarded, and the plate was air-dried again. Biofilms were stained with crystal violet for 5 min and washed three times with ddH_2_O to remove excess dye. Finally, 200 μL of 95% ethanol was added to each well to solubilize the biofilm-bound crystal violet, and the absorbance was measured at 595 nm using a microplate reader. This experiment was repeated three times.

### Outer membrane permeability assay

The outer membrane permeability was measured using the 1-N-phenylnaphthylamine (NPN) probe method, as previously described (Helander and Mattila-Sandholm, 2000). The WT-P2C, Δ*vscW*-P2C, and CΔ*vscW*-P2C strains were cultured in sterile TSB broth at 28 °C and 180 rpm for 24 h. The cultures were centrifuged at 6,000 rpm for 5 min, and the cell pellets were collected, washed three times with 1× phosphate-buffered saline (PBS), and resuspended to an OD_600_ of 0.5. Aliquots (190 μL) of bacterial suspensions were transferred to opaque 96-well plates. The NPN stock solution (10 mmol/L) was diluted 10-fold in DMSO to obtain a 1 mmol/L intermediate solution, which was further diluted 10-fold in PBS to obtain a working solution of 100 μmol/L. Subsequently, 10 μL of the NPN working solution was added to each well containing 190 μL of the bacterial suspension, resulting in a final NPN concentration of 5 μmol/L. The plates were gently shaken to mix the reaction system, and fluorescence intensity was measured using a multimode microplate reader (SpectraMax i3x, Molecular Devices, Shanghai, China) at excitation and emission wavelengths of 350 and 420 nm, respectively. Fluorescence was recorded every 2 min until a stable plateau was reached. Ten technical replicates were prepared for each group in each experiment. The experiment was independently repeated three times.

### Antimicrobial susceptibility testing

The antimicrobial susceptibilities of the WT-P2C and Δ*vscW*-P2C strains were evaluated using the Kirby-Bauer disk diffusion method. The cultures were diluted to 1 × 10^6^ CFU/mL in sterile TSB and an appropriate volume of the bacterial suspension was spread evenly over the surface of the TSB agar. After the agar plates were fully absorbed, antimicrobial susceptibility disks were placed on the agar, and the plates were incubated at 28 °C for 24 h before measuring the inhibition zone diameters. Susceptibility categories were assigned according to the antimicrobial susceptibility testing standards of the Clinical and Laboratory Standards Institute (CLSI). The experiment was performed in triplicate.

### Proteomic analysis

WT-P2C and Δ*vscW*-P2C cells cultured in the same growth phase were collected from three independent biological replicates per group. After washing with pre-chilled PBS, the cells were rapidly frozen and sent to Shanghai Majorbio Biopharm Technology Co., Ltd. for library-free, data-independent acquisition (DIA) quantitative proteomic analysis. After protein extraction, BCA quantification, reduction, alkylation, trypsin digestion, peptide desalting, and quantification, the samples were separated using a Vanquish Neo nano-flow liquid chromatography system and analyzed using an Orbitrap Astral mass spectrometer in DIA mode. Raw data were imported into Spectronaut 19 and searched against an in-house database constructed from the predicted protein sequences in the *V. parahaemolyticus* P2C genome. The false discovery rate at both the peptide and protein levels was controlled at ≤1%. Protein abundance was quantified in a label-free manner using the Maxquant Label-free Quantification (MaxLFQ) algorithm. Differentially expressed proteins (DEPs) were identified using P < 0.05, fold change [FC] > 1.2 or FC < 0.833 as the criteria. DEPs were functionally annotated using the GO, KEGG, Pfam, Swiss-Prot, NR, and eggNOG databases, and related analyses were performed on the Majorbio Cloud Platform.

### RT-qPCR

Total RNA was extracted from WT-P2C and Δ*vscW*-P2C cells using an RNA extraction kit (Vazyme Biotech Co., Ltd., Nanjing, China) and cDNA was synthesized using a reverse transcription kit. RT-qPCR was performed using Taq Pro Universal SYBR qPCR Master Mix. The cycling conditions were 95 °C for 30 s, followed by 40 cycles of 95 °C for 15 s, and 60 °C for 30 s. *recA* was used as the reference gene. The assayed genes included those associated with the T3SS basal structure (*vscC* and *vscJ*), needle structure and needle-length control (*vscF* and *vscP*), inner membrane export apparatus (*vscV* and *vscT*), and ATPase complex and secretion platform (*vscN*, *vscL*, and *vscO*). All primers were designed based on the WT-P2C genome, and their specificity was verified by agarose gel electrophoresis and melting-curve analysis. Relative expression was calculated using the 2^−ΔΔ^Ct method. The experiment was repeated three times, and the primer information is provided in **Table 3**.

**Table 3:** Primer sequences used for RT-qPCR detection of T3SS- and T6SS-related genes in WT-P2C and *ΔvscW*-P2C.

Genes	Sequences (5′–3′)	Product length (bp)	Source=
*vscC*	F:GCCACGTTACGTTGAGTTAGTAGTG	198	This study
	R:CCAACCACACGGCTCAAT		
*vscF*	F:AGTGTAAACCTCGATGACGTTAAAAC	209	This study
	R:ATCGACTGCATCACATCTTTGAT		
*vscJ*	F:CGCAAAATCGCAAGAGTTGTC	187	This study
	R:CAATTTGATTTGAGGCACGTAAGAG		
*vscL*	F:TCATTTGTTGAAATAAAAACCGACAACT	199	This study
	R:CCTGCTCCAAACCATCTTGATAG		
*vscN*	F:CAAGTCGAGTGATGAACCAGATT	201	This study
	R:GCTCATGTGTGCCTTGACGTAA		
*vscO*	F:CGCTCGGTAGCGGACTATC	198	This study
	R:TAGCAGAGTGCGCTCTTGTTC		
*vscP*	F:CCACAATCTCCAATGCCG	206	This study
	R:GTCTTACGTTGGTTGCTACTTGTC		
*vscT*	F:GGCTCATGGCGTGCTTTATC	193	This study
	R:AGCATACCCAGCAAGACTTCTT		
*vscV*	F:GATCGGCGCAAACATGAGTAT	207	This study
	R:CCGCCAACGACGAAGTTAC		
*vscW*	F:CCAAGCCCTTGATAGCTGTGTAC	190	This study
	R:AGCACTTATTGGCGCTGATTG		
*recA*	F:GCTAGTAGAAAAAGCGGGTG	165	(Ma et al., 2015)
	R:GCAGGTGCTTCTGGTTGAG		
*gene1416/hcp*	F:CCGTCCACTTGTTGTGACTA	130	This study
	R:CTCTTGCTTGCCTTGAACTG		
*gene1415/vasG*	F:GTGTGCACGAGTGAACATCA	130	This study
	R:CTTGTCGCCAGTTTGTTGCA		
*gene1424/impB*	F:GACGCCAATCGAAGAACGTA	130	This study
	R:GATCTGTGCGCCTTCTTCAT		
*gene1425/impC*	F:GTGGCTTACGTGAACACGAA	130	This study
	R:CTATCTGTTACGTGCTCGTC		
*gene1430/impL*	F:CGACCAAGCGAGATATTCAG	130	This study
	R:GGTGAGAATTGAGTGCGGAA		
*gene4675/hcp*	F:CCGATTGTTGCTTCCTTG	187	This study
	R:CGACAGCGGAACAATATAAGGA		
*gene4682/impC*	F:AACAACTCGATCCTACGGTG	129	This study
	R:CCTTCATCGCTTCTTCAGTC		
*gene4683/impB*	F:TCGTCATTTGCTAACTTGTTTG	170	This study
	R:CCTTTTGTGGTGGGTGTTATG		

To validate the proteomic results, eight genes corresponding to differentially expressed T6SS-related proteins were selected for RT-qPCR analysis. These included five upregulated genes (gene1416/*hcp*, gene1415/*vasG*, gene1424/*impB*, gene1425/*impC*, and gene1430/*impL*) and three downregulated genes (gene4675/hcp, gene4682*/impC*, and gene4683/*impB*). All primers were designed based on the WT-P2C genome sequence and screened for target locus specificity. Agarose gel electrophoresis confirmed the presence of a single amplification product for each target, enabling locus-specific detection of homologous genes. RNA extraction, cDNA synthesis, reaction mixture preparation, and amplification were performed as described previously. Relative expression was calculated using the 2^−ΔΔ^Ct method and then transformed to log_2_ fold change for comparison with the proteomic results. Three independent biological replicates were used and the primers used are listed in **Table 3**.

### Pathogenicity assay

Healthy Pacific white shrimp (8 ± 1 g) were randomly divided into unchallenged control, WT-P2C challenge, and *ΔvscW*-P2C challenge groups. Each group consisted of 24 shrimp housed in three replicate tanks. Water temperature and salinity were maintained at 28 °C and 28‰, respectively. Shrimp were immersed in sterile PBS or in WT-P2C or Δ*vscW*-P2C suspensions at 4 × 10^7^ CFU/mL. The shrimp remained in the respective treatment water from the start of the challenge until sampling. Mortality was recorded for seven consecutive days following the challenge. Survival curves were generated using the Kaplan–Meier method. All animal procedures strictly complied with the Regulations for the Administration of Affairs Concerning Experimental Animals and Technical Specifications for Aquatic Laboratory Animals. Discarded tissues and culture wastewater were sterilized and disposed according to medical waste standards, and the experimental premises and equipment were disinfected with sodium hypochlorite solution (100 mg/L).

### Assay of immune-related enzyme activities in the hepatopancreas of Pacific white shrimp

To compare the changes in hepatopancreatic immune-related enzyme activities among the unchallenged control, WT-P2C-challenged, and *ΔvscW*-P2C-challenged groups, three Pacific white shrimp were randomly selected from each group at 0, 24, 48, and 72 h post-challenge, and hepatopancreatic tissues were collected. The samples were immediately snap-frozen in liquid nitrogen and transferred to a -80 °C freezer for storage. The activities of alkaline phosphatase (AKP), acid phosphatase (ACP), superoxide dismutase (SOD), and lysozyme (LZM) in hepatopancreatic tissue were measured using a multimode microplate reader. Three biological replicates were included in each treatment group for analysis.

### Analysis of immune-related gene expression in Pacific white shrimp

To investigate the effects of the *ΔvscW* mutant and wild-type (WT-P2C) on immune-related gene expression in Pacific white shrimp, hepatopancreatic tissues were collected at 0, 24, 48, and 72 h post-challenge for subsequent analysis. Total tissue RNA was extracted according to the manufacturer’s instructions using an RNA extraction kit (Vazyme Biotech Co., Ltd., Nanjing, China), and complementary DNA (cDNA) was synthesized via reverse transcription. The RT-qPCR reaction mixture, instrument, and amplification program are described in the RT-qPCR subsection above. L21 was used as the reference gene, and the assayed immune-related genes included lysozyme, Toll receptor, crustin, TCTP, and penaeidin 4. The primers used for immune-related gene expression analysis are listed in **Table 4**.

**Table 4:** Primers used for RT-qPCR analysis of immune-related genes in *Litopenaeus vannamei*.

Genes	Sequences (5′–3′)	Source
*Lysozyme*	F:GAAGCGACTACGGCAAGAAC R:AACCGTGAGACCAGCACTCT	(Wang et al., 2010)
*Toll receptor*	F:ATGTGCGTGCGGATACATTA R:GGGTGTTGGATGTCGAGAGT	(Wang et al., 2010)
*Crustin*	F:GAGGGTCAAGCCTACTGCTG R:ACTTATCGAGGCCAGCACAC	(Wang et al., 2010)
*TCTP*	F:CAATGGACCCTGATGGC R:GCTTCTCCTCTGTTAGACCGTAT	(Wu et al., 2013)
*Penaeidin4*	F:GCCCGTTACCCAAACCATC R:CCGTATCTGAAGCAGCAAAGTC	(Wang et al., 2010)
*L21*	F:GTTGACTTGAAGGGCAATG R:CAATGAGAGCTTGCCTTTCC	(Stephens et al., 2012)

### Data analysis

Data were organized using Microsoft Excel and statistically analyzed using GraphPad Prism version 9.0. Statistical differences were assessed using one-way or two-way analysis of variance (ANOVA), followed by Tukey’s multiple comparison test. The results are presented as mean ± standard deviation (mean ± SD). Independent-samples t-tests were used to analyze the differences in diameter and absorbance. All experiments were performed independently at least three times. Statistical significance was set at P < 0.05. In the figures, * indicates P < 0.05, ** indicates P < 0.01, and ns indicates no significant difference.

## Results

### Construction and identification of the *vscW* deletion mutant and complemented strain

The *vscW* deletion mutant was constructed using homologous recombination. Using WT-P2C genomic DNA as a template, the upstream and downstream homologous arms of *vscW* were amplified with *vscW*-1F/*vscW*-1R and *vscW*-2F/*vscW*-2R, yielding 506- and 493-bp fragments, respectively. The two homologous arms were ligated into the suicide plasmid pRE112 to generate pRE112-UD*vscW* plasmids. PCR with pRE112-F/pRE112-R yielded a 1,194-bp product, confirming the successful introduction of the recombinant plasmid into the donor strains. Candidate deletion mutants were obtained by conjugative transfer and sucrose counter-selection. PCR with the internal primers in-*vscW*-F/in-*vscW*-R yielded the expected 240-bp deletion product (**Figure 1A**). Further verification with *vscW*-1F/*vscW*-2R yielded 1,991- and 999-bp products from WT-P2C and *ΔvscW*-P2C, respectively, confirming the successful construction of the deletion mutants (**Figure 1B**).

**Figure 1:**
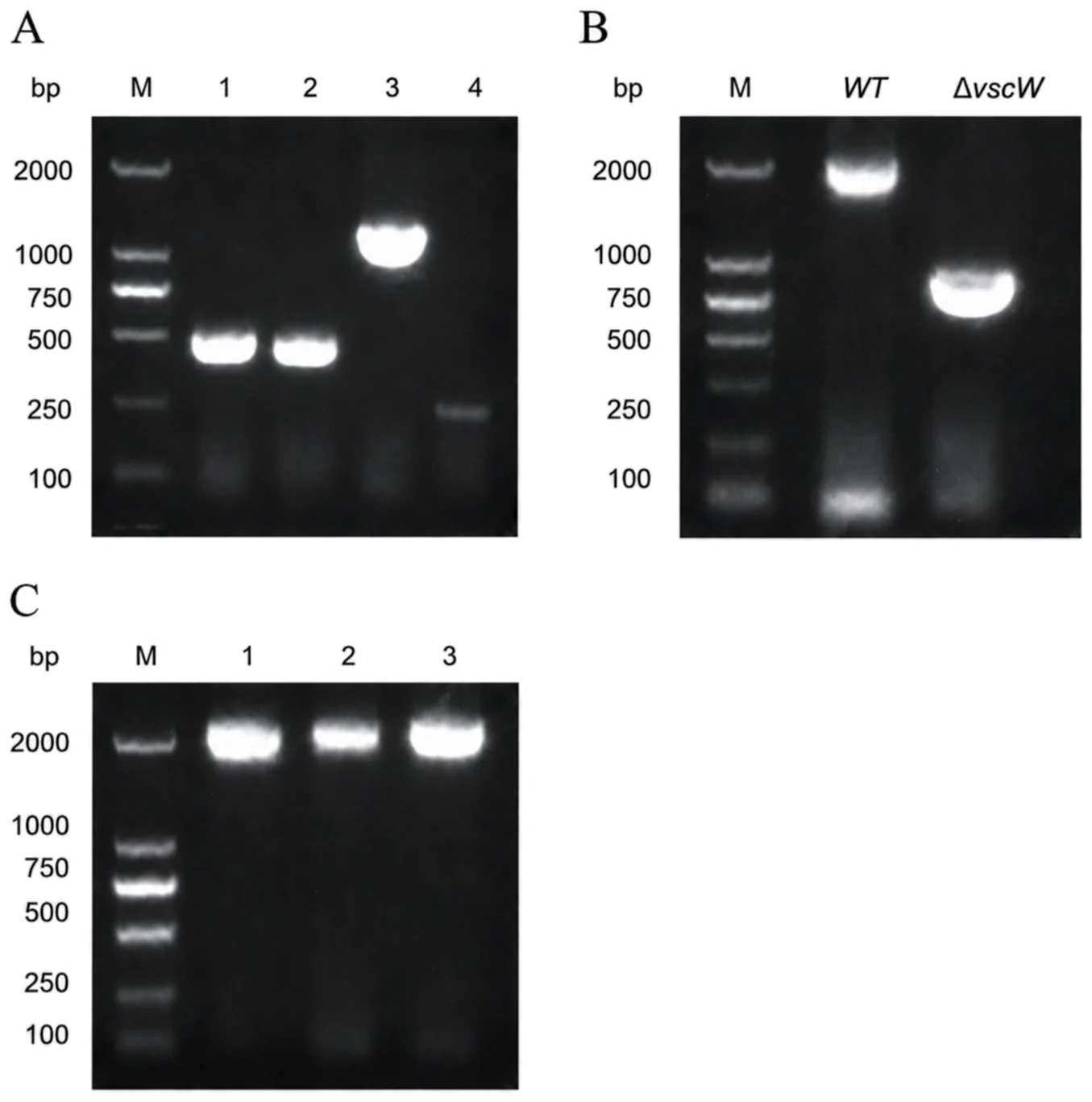
PCR identification of the *vscW* deletion mutant and complemented strain. M, DL2000 DNA marker. **(A)** PCR identification of the *vscW* deletion mutant. Lane 1, upstream homologous arm of *vscW*; lane 2, downstream homologous arm of *vscW*; lane 3, recombinant plasmid pRE112-UD*vscW*; lane 4, identification of *ΔvscW*-P2C using in-*vscW*-F/in-*vscW*-R primers. **(B)** PCR amplification across the deleted region. Lane 1, WT-P2C; lane 2, *ΔvscW*-P2C. **(C)** PCR identification of the complementary strain. Lane 1, *vscW* complementation fragment; lane 2, recombinant plasmid pBBR1-P+*vscW*; lane 3, complemented strain C*ΔvscW*-P2C.

WT-P2C genomic DNA was used as a template, and a 1,983-bp *vscW* complementation fragment was amplified using *the vscW*-HF/*vscW*-HR primers. This fragment was ligated into pBBR1MCS-5 to generate the recombinant plasmid pBBR1-P+*vscW*. The recombinant plasmid was transferred into *ΔvscW*-P2C by conjugation. PCR screening of colonies positive for pBBR1-F/pBBR1-R yielded the expected 2,153-bp product. These results confirmed the successful introduction of the complementation plasmid into the deletion mutant and the generation of C*ΔvscW*-P2C (**Figure 1C**).

### *vscW* deletion did not markedly affect *V. parahaemolyticus* P2C growth

To evaluate whether *vscW* deletion influenced P2C growth, the growth curves of WT-P2C, *ΔvscW*-P2C, and C*ΔvscW*-P2C were measured under the same culture conditions as described above. All three strains grew rapidly from 2 to 6 h before gradually reaching the stationary phase. Their overall growth patterns were similar, indicating that *vscW* deletion had no detectable effect on P2C growth under the tested conditions (**Figure 2**).

**Figure 2:**
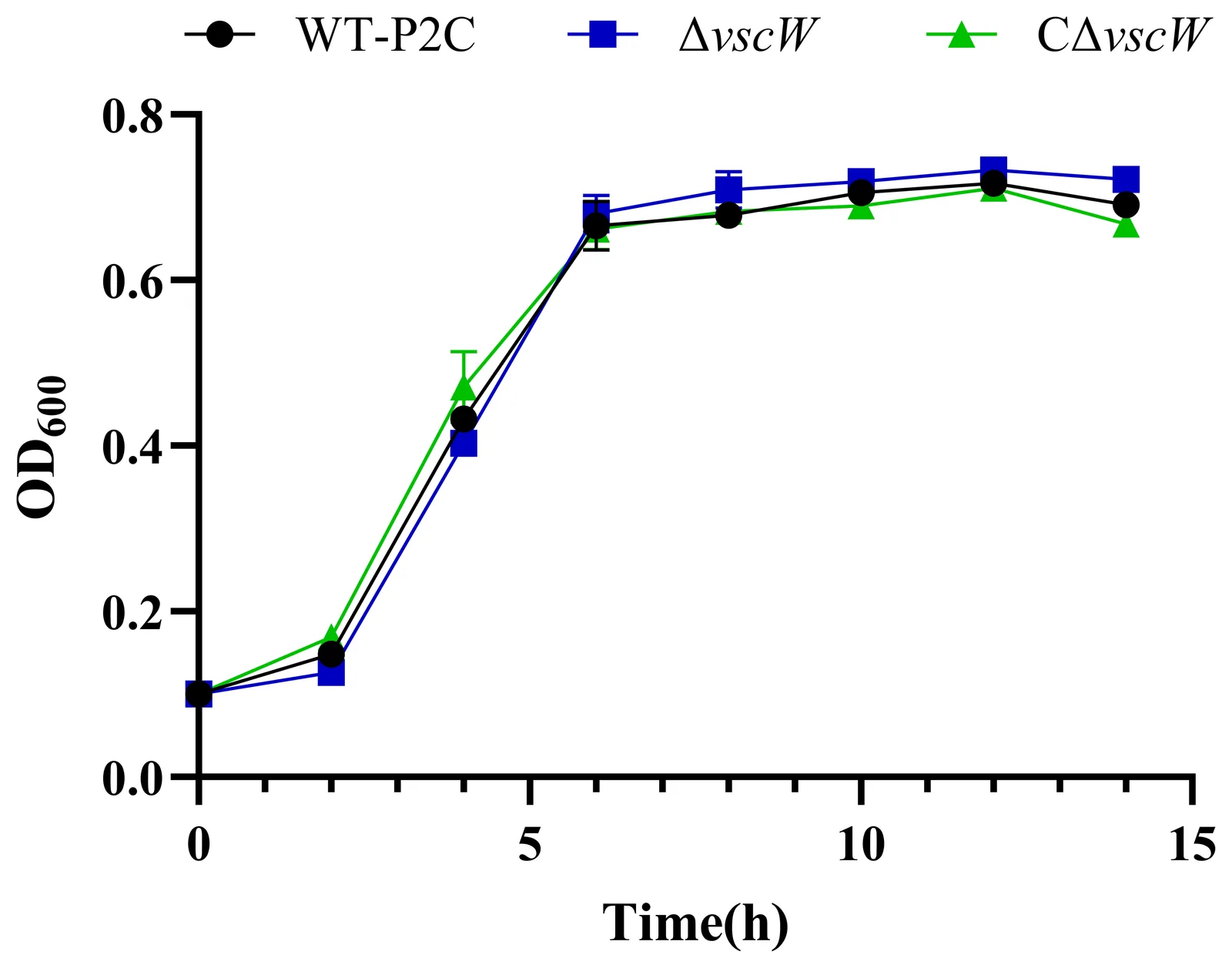
Growth curves for WT-P2C, *ΔvscW*-P2C, and C*ΔvscW*-P2C. The OD_600_ values of the three strains were measured at 2-h intervals during incubation at 28 °C under identical culture conditions. Data are presented as the mean ± SD (n = 3 biological replicates).

### *vscW* deletion affected *V. parahaemolyticus* P2C motility

Transmission electron microscopy was used to examine the morphology and flagellar structure of WT-P2C and *ΔvscW*-P2C cells. Except for fewer visible flagella in Δ*vscW*-P2C, the two strains exhibited similar overall cell morphology; this flagellar difference may be associated with the reduced motility of the mutant (**Figures 3A, B**). Observation of the strains inoculated onto TSB semi-solid media containing 0.3% or 0.6% agar for 12 h showed that *ΔvscW*-P2C exhibited reduced swimming motility compared with WT-P2C. The complemented strain partially restored the swimming phenotype, but remained different from that of WT-P2C, whereas no apparent difference in swarming motility was observed (**Figure 3C**). Quantification of the motility-zone diameters showed that the swimming-zone diameters of WT-P2C and *ΔvscW*-P2C on 0.3% agar were 14.77 ± 0.31 and 8.70 ± 0.26 mm, respectively, representing a reduction of approximately 41.1% in *ΔvscW*-P2C relative to WT-P2C. The corresponding diameters of WT-P2C and C*ΔvscW*-P2C were 15.16 ± 0.47 and 12.70 ± 0.34 mm, respectively (P < 0.01). On 0.6% agar, the swarming-zone diameters of WT-P2C and *ΔvscW*-P2C were 14.10 ± 0.40 and 13.93 ± 0.75 mm, respectively, with no significant difference between the two strains (P > 0.05; **Figure 3D**).

**Figure 3:**
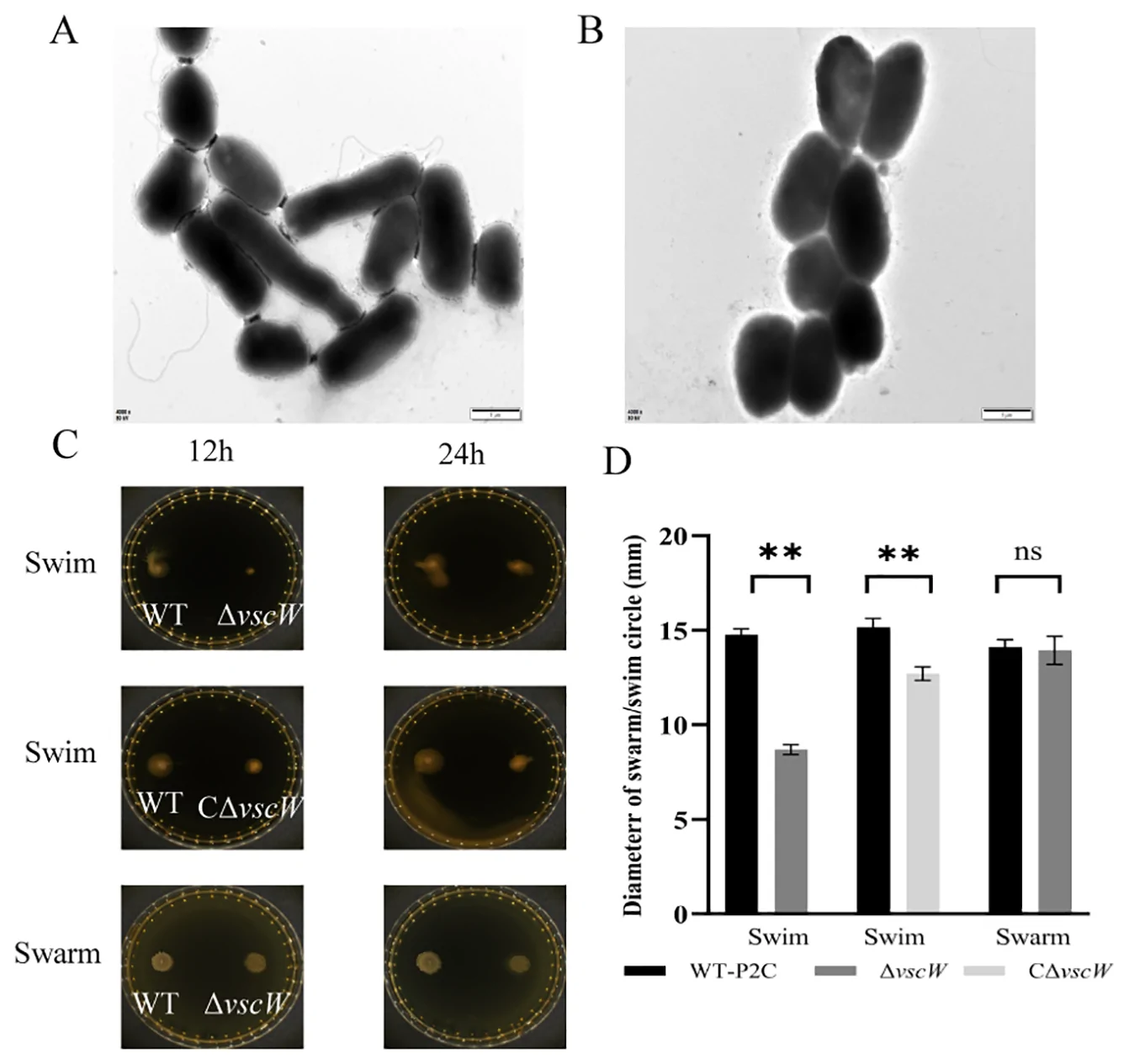
Effects of *vscW* deletion on the morphology and motility of *V. parahaemolyticus* P2C. **(A, B)** Transmission electron micrographs showing the cellular morphology and flagellar structures of WT-P2C **(A)** and *ΔvscW*-P2C **(B)**. Scale bars, 1 μm. **(C)** Representative swimming and swarming phenotypes of WT-P2C, *ΔvscW*-P2C, and C*ΔvscW*-P2C cells after incubation at 28 °C for 12 h and 24 h. Swimming and swarming motilities were evaluated on TSB plates containing 0.3% and 0.6% agar, respectively. **(D)** Quantification of swimming and swarming zone diameters after 24 h of incubation. In each independent experiment, each migration-zone diameter was measured three times. Data are presented as the mean ± SD from three independent experiments (n = 3 biological replicates). **P < 0.01; ns, not significant.

### *vscW* deletion reduced biofilm formation and increased outer membrane permeability

Biofilm formation by WT-P2C, *ΔvscW*-P2C, and C*ΔvscW*-P2C was quantified by a microtiter crystal violet staining assay. The OD_595_ values of WT-P2C and *ΔvscW*-P2C were 1.40 ± 0.13 and 0.77 ± 0.12, respectively. Compared with that of WT-P2C, the OD_595_ of *ΔvscW*-P2C decreased by approximately 45% (P < 0.01), indicating that *vscW* deletion reduced the biofilm-forming ability of this strain. The OD_595_ of C*ΔvscW*-P2C returned to a level close to that of WT-P2C (P > 0.05), suggesting that *vscW* complementation partially restored this phenotype (**Figure 4A**).

**Figure 4:**
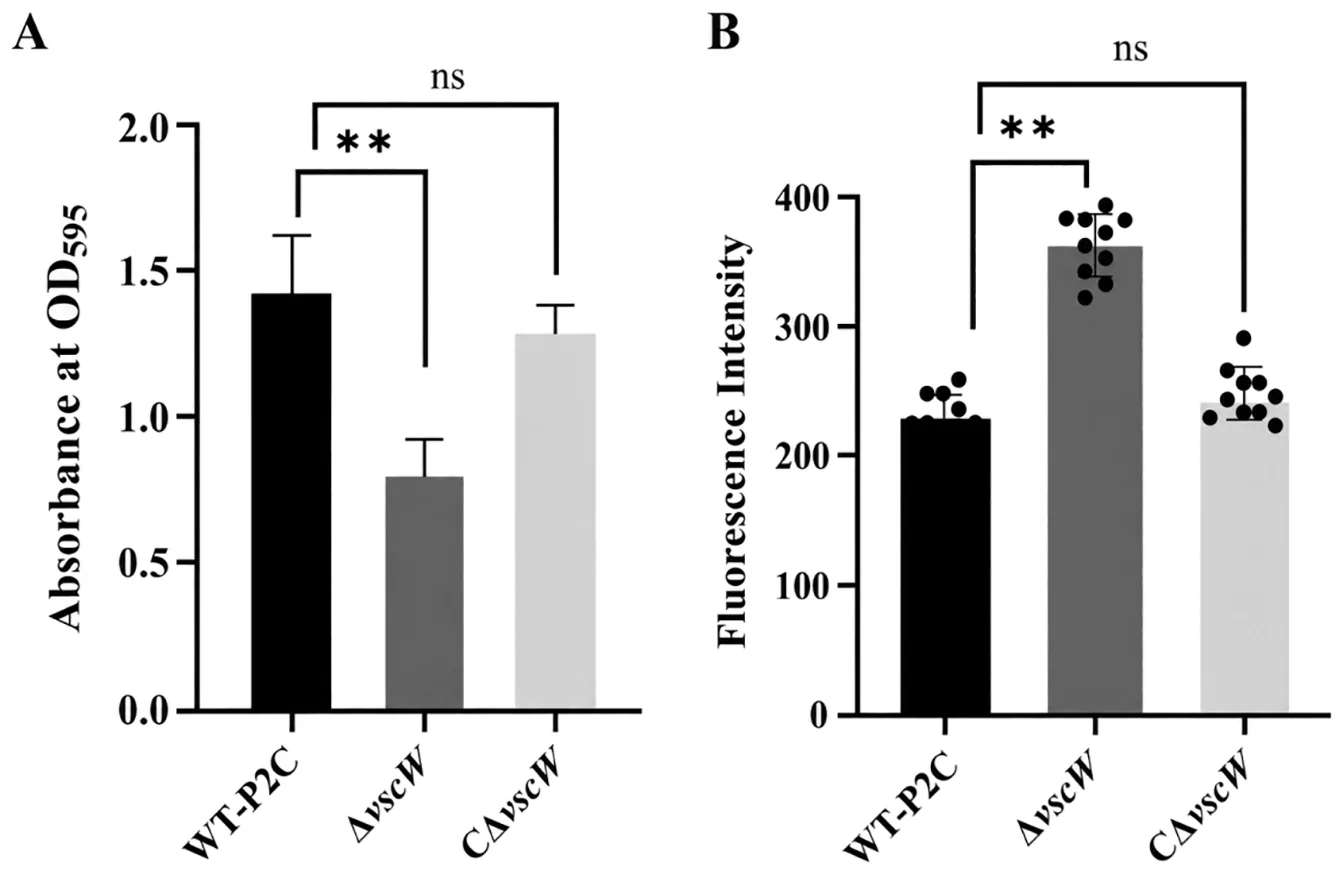
Effects of *vscW* deletion on biofilm formation and outer membrane permeability of P2C. **(A)** Biofilm formation by WT-P2C, *ΔvscW*-P2C, and C*ΔvscW*-P2C, quantified by OD_595_; and **(B)** NPN uptake by the three strains. Fluorescence was measured at excitation and emission wavelengths of 350 and 420 nm, respectively. All three strains were cultured at 28 °C. Data are presented as the mean ± SD (n = 3 biological replicates). ** indicates P < 0.01; ns, not significant.

The NPN uptake assay showed that the fluorescence intensity was significantly higher in *ΔvscW*-P2C than in WT-P2C (P < 0.01), indicating that *vscW* deletion increased outer membrane permeability. The fluorescence intensity of C*ΔvscW*-P2C returned to a level close to that of WT-P2C (P > 0.05). Overall, *vscW* deletion reduced biofilm formation and altered the outer membrane barrier properties of P2C cells (**Figure 4B**).

### Differences in antimicrobial susceptibility

The susceptibility of WT-P2C and *ΔvscW*-P2C to the 36 antimicrobial agents is shown in **Table 5**. Both strains were susceptible to 22 agents, including furazolidone, florfenicol, enrofloxacin, chloramphenicol, ceftazidime, ceftriaxone, quinolones, and aminoglycosides. Both strains were classified as intermediate for pipemidic acid and azithromycin and were resistant to 12 agents, including penicillin, oxacillin, ampicillin, cefazolin, cephalexin, cefradine, erythromycin, streptomycin, and rifampin.

**Table 5:** Antimicrobial susceptibility profiles of WT-P2C and *ΔvscW*-P2C.

No.	Antimicrobial agent	Disk content (µg/disc)	Interpretive criteria		Inhibition zone (mm)		Interpretation	
			R	S	WT-P2C	Δ*vscW*-P2C	WT-P2C	*ΔvscW*-P2C
1	Furazolidone	30	≤ 14	≥ 17	21.83 ± 0.29	20.63 ± 0.78	S	S
2	Florfenicol	30	≤ 12	≥ 18	24.67 ± 0.58	22.83 ± 0.76	S	S
3	Enrofloxacin	10	≤ 15	≥ 19	24.83 ± 0.76	34.33 ± 0.58	S	S
4	Chloramphenicol	30	≤ 12	≥ 18	22.50 ± 0.50	24.00 ± 0.73	S	S
5	Cefazolin	30	≤ 14	≥ 18	9.73 ± 0.25	8.50 ± 0.87	R	R
6	Cephalexin	30	≤ 14	≥ 18	0 ± 0	5.55 ± 0.33	R	R
7	Ampicillin	10	≤ 13	≥ 17	0 ± 0	0 ± 0	R	R
8	Oxacillin	1	≤ 14	≥ 20	0 ± 0	0 ± 0	R	R
9	Penicillin	10	≤ 11	≥ 15	0 ± 0	0 ± 0	R	R
10	Cefradine	30	≤ 14	≥ 18	8.63 ± 0.40	9.83 ± 0.29	R	R
11	Ceftazidime	30	≤ 17	≥ 21	30.57 ± 0.51	31.67 ± 0.89	S	S
12	Ceftriaxone	30	≤ 19	≥ 23	33.49 ± 0.33	33.37 ± 0.36	S	S
13	Cefotaxime	30	≤ 22	≥ 26	31.33 ± 0.34	31 ± 0.73	S	S
14	Ceftizoxime	30	≤ 21	≥ 25	31.67 ± 0.39	30.67 ± 0.38	S	S
15	Norfloxacin	10	≤ 12	≥ 17	26.67 ± 0.3	26.33 ± 0.21	S	S
16	Ofloxacin	5	≤ 12	≥ 16	24.67 ± 0.52	32.00 ± 0.46	S	S
17	Erythromycin	15	≤ 13	≥ 23	10.83 ± 0.4	10.00 ± 0.72	R	R
18	Minocycline	30	≤ 12	≥ 16	23.47 ± 0.11	25.00 ± 0.12	S	S
19	Doxycycline	30	≤ 10	≥ 14	18.35 ± 0.64	23.33 ± 0.55	S	S
20	Tetracycline	30	≤ 11	≥ 15	21.07 ± 0.81	22.87 ± 1.03	S	S
21	Neomycin	30	≤ 17	≥ 23	16.00 ± 0.80	16.20 ± 0.70	R	R
22	Pipemidic acid	30	≤ 22	≥ 28	22.77 ± 0.75	27.17 ± 0.76	I	I
23	Nalidixic acid	30	≤ 13	≥ 19	32.67 ± 0.58	33.83 ± 0.29	S	S
24	Azithromycin	15	≤ 13	≥ 18	16.77 ± 0.68	14.33 ± 0.76	I	I
25	Clarithromycin	15	≤ 13	≥ 18	8.70 ± 0.72	11.37 ± 0.81	R	R
26	Acetylspiramycin	30	≤ 13	≥ 18	0.00 ± 0.00	0.00 ± 0.00	R	R
27	Streptomycin	10	≤ 11	≥ 15	0.00 ± 0.00	0.00 ± 0.00	R	R
28	Kanamycin	30	≤ 13	≥ 18	20.50 ± 0.87	23.80 ± 0.61	S	S
29	Gentamicin	10	≤ 12	≥ 15	15.33 ± 0.58	20.50 ± 0.87	S	S
30	Amikacin	30	≤ 13	≥ 18	24.40 ± 0.66	28.67 ± 1.04	S	S
31	Cefoperazone-sulbactam	75/75	≤ 15	≥ 21	28.87 ± 0.40	30.33 ± 0.58	S	S
32	Rifampin	5	≤ 16	≥ 20	5.17 ± 0.29	11.17 ± 0.76	R	R
33	Polymyxin B	300IU	≤ 11	≥ 12	12.07 ± 0.93	13.57 ± 0.86	S	S
34	Fleroxacin	5	≤ 15	≥ 19	30.33 ± 0.58	34.50 ± 0.87	S	S
35	Ciprofloxacin	5	≤ 15	≥ 21	27.63 ± 1.55	31.00 ± 1.00	S	S
36	Lomefloxacin	10	≤ 18	≥ 22	32.17 ± 0.76	39.37 ± 0.65	S	S

R, resistant; I, intermediate; S, susceptible. Data are presented as the mean ± SD (n = 3).

Deletion of *vscW* did not alter the susceptibility, intermediate, or resistance classifications of P2C for the tested drugs. However, the inhibition zone diameters were larger for *ΔvscW*-P2C than for WT-P2C for enrofloxacin, ofloxacin, doxycycline, gentamicin, amikacin, fleroxacin, and lomefloxacin, suggesting that *vscW* deletion may affect the degree of susceptibility to some agents; however, these differences were insufficient to alter the categorical interpretations.

### *vscW* deletion altered the transcription of T3SS-related genes

RT-qPCR was performed to compare the transcription levels of T3SS-related genes in WT-P2C and *ΔvscW*-P2C. As shown in **Figure 5**, the expression levels of *vscJ* and *vscF* did not differ significantly between the two strains. In *ΔvscW*-P2C, *vscL*, *vscO*, and *vscT* were significantly downregulated, whereas *vscC*, *vscN*, *vscP*, and *vscV* were significantly upregulated (P < 0.01). These results indicated that *vscW* deletion altered the transcription of multiple T3SS-related genes.

**Figure 5:**
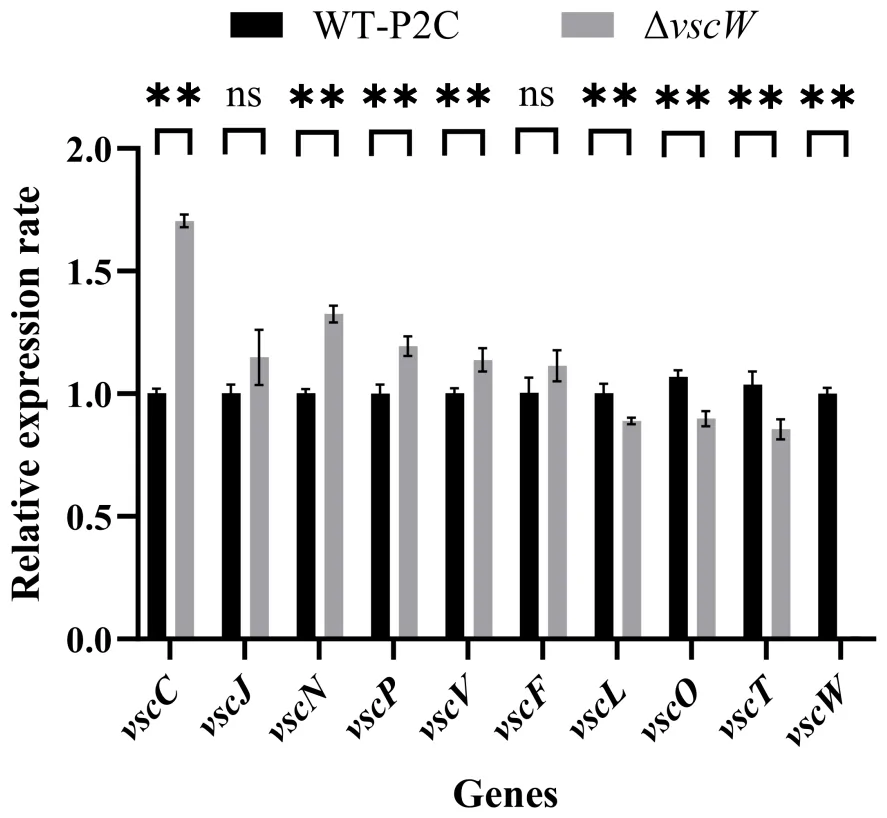
Effects of *vscW* deletion on transcription of T3SS-related genes in P2C. The expression levels of the target genes were normalized to *recA*, with WT-P2C used as the calibrator, and calculated using the 2^^−ΔΔ^Ct method. Data are presented as the mean ± SD (n = 3 biological replicates). * indicates P < 0.05; ** indicates P < 0.01; ns, not significant.

### Effect of *vscW* deletion on protein expression in *V. parahaemolyticus* P2C

Sample correlation analysis and principal component analysis (PCA) were performed to assess global differences in protein expression between WT-P2C and *ΔvscW*-P2C (**Figures 6A, C**). The two groups were clearly separated along PC1, which explained 86.40% of the total variance, indicating distinct overall protein expression patterns among the groups. A total of 3,851 proteins were detected across the two groups, of which 243 were differentially expressed, including 130 upregulated and 113 downregulated proteins (**Figure 6B**). KEGG functional classification indicated changes in T3SS-related proteins and more pronounced alterations in T6SS-related proteins, including Hcp, VgrG, VasG, ImpB, ImpC, ImpL, and DotU (**Figures 6D, E**). Proteins linked to membrane transport, two-component regulation, c-di-GMP signaling, biofilm formation, flagellar motility, and chemotaxis were also altered. Overall, the differentially expressed proteins were primarily associated with bacterial secretion systems, membrane transport, motility, chemotaxis, and environmental adaptation.

**Figure 6:**
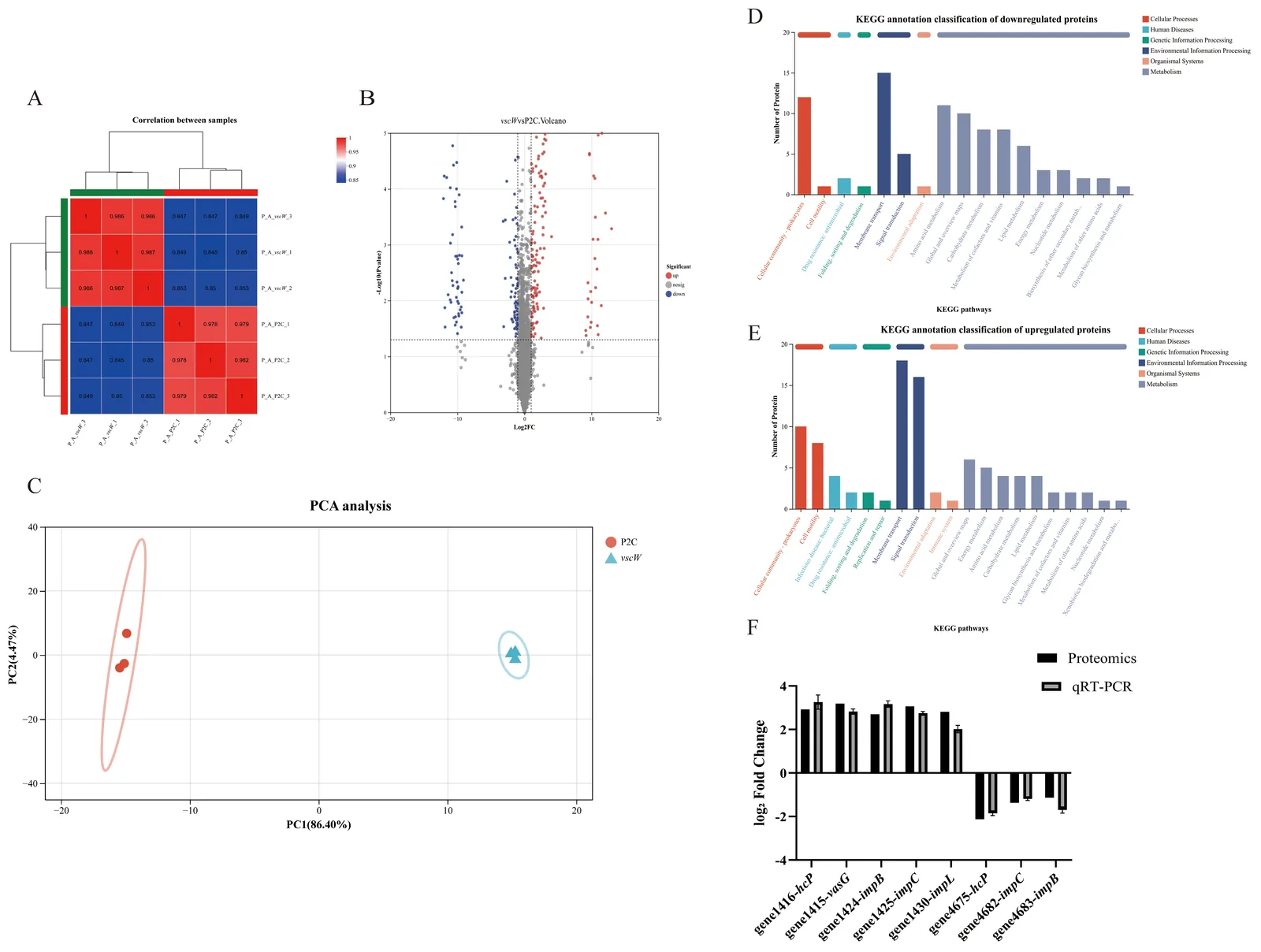
Proteomic analysis of WT-P2C and *ΔvscW*-P2C. **(A)** Pearson’s correlation heatmap of samples; **(B)** volcano plot of differentially expressed proteins between Δ*vscW*-P2C and WT-P2C. Red, blue, and gray dots represent significantly upregulated, significantly downregulated, and non-differentially expressed proteins, respectively; **(C)** principal component analysis (PCA) of the proteomic profiles of WT-P2C and Δ*vscW*-P2C; **(D)** KEGG functional classification of downregulated proteins; **(E)** KEGG functional classification of upregulated proteins; and **(F)** comparison of proteomic and RT-qPCR results for selected T6SS-related targets. Black bars represent proteomic results, whereas gray bars represent RT-qPCR results. Both datasets are presented as log_2_ fold changes relative to WT-P2C. Data are presented as the mean ± SD (n = 3 biological replicates).

The expression trends of the selected T6SS-related genes were generally consistent with the corresponding changes in protein abundance, supporting the reliability of our proteomic results (**Figure 6F**).

### *vscW* deletion reduced *V. parahaemolyticus* P2C pathogenicity in Pacific white shrimp

Shrimp were monitored for seven days after the immersion challenge, and the final survival rates were 33.3% and 58.3% in the WT-P2C and *ΔvscW*-P2C groups, respectively. Compared with that in the WT-P2C group, the final mortality in the *ΔvscW*-P2C group was reduced by approximately 37.5% (**Figure 7A**).

**Figure 7:**
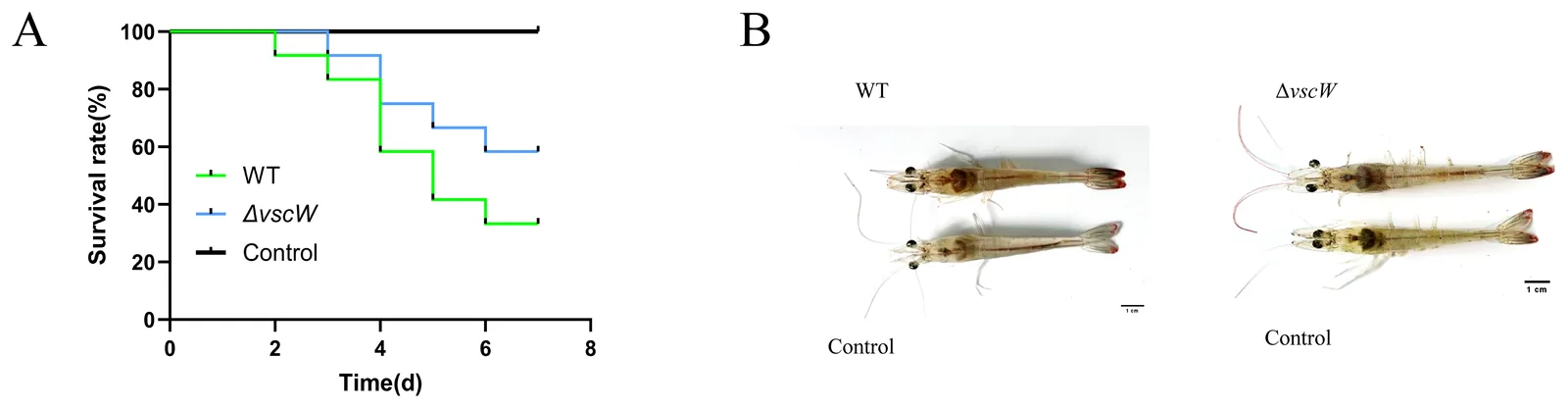
Pathogenicity of WT-P2C and Δ*vscW*-P2C in Pacific white shrimp (*Litopenaeus vannamei*). **(A)** Survival curves of *L. vannamei* in the uninfected control, WT-P2C-infected, and Δ*vscW*-P2C-infected groups (n = 24 shrimp per group; three replicate tanks per group). Survival curves were compared using the log-rank (Mantel–Cox) test. **(B)** Representative gross signs of shrimp in each group at 7 d post-challenge. Scale bars, 1 cm.

The shrimp challenged with WT-P2C exhibited reddening of the body and tail fan, disrupted intestinal continuity, and an abnormal hepatopancreatic appearance (**Figure 7B**). These gross changes were milder in shrimp challenged with *ΔvscW*-P2C.

Immune-related gene analysis indicated that before the P2C challenge, the relative expression levels of *lysozyme*, *toll receptor*, *crustin*, *TCTP*, and *penaeidin 4* did not differ significantly between the groups. After the challenge, *crustin* showed a strong response at 24 h, whereas *lysozyme*, *toll receptor*, *TCTP*, and *penaeidin 4* reached higher expression levels at 48 h post-challenge. The induction of these genes was generally lower in the *ΔvscW*-P2C group than in the WT-P2C group, particularly at 24–48 h post-challenge (**Figure 8**).

**Figure 8:**
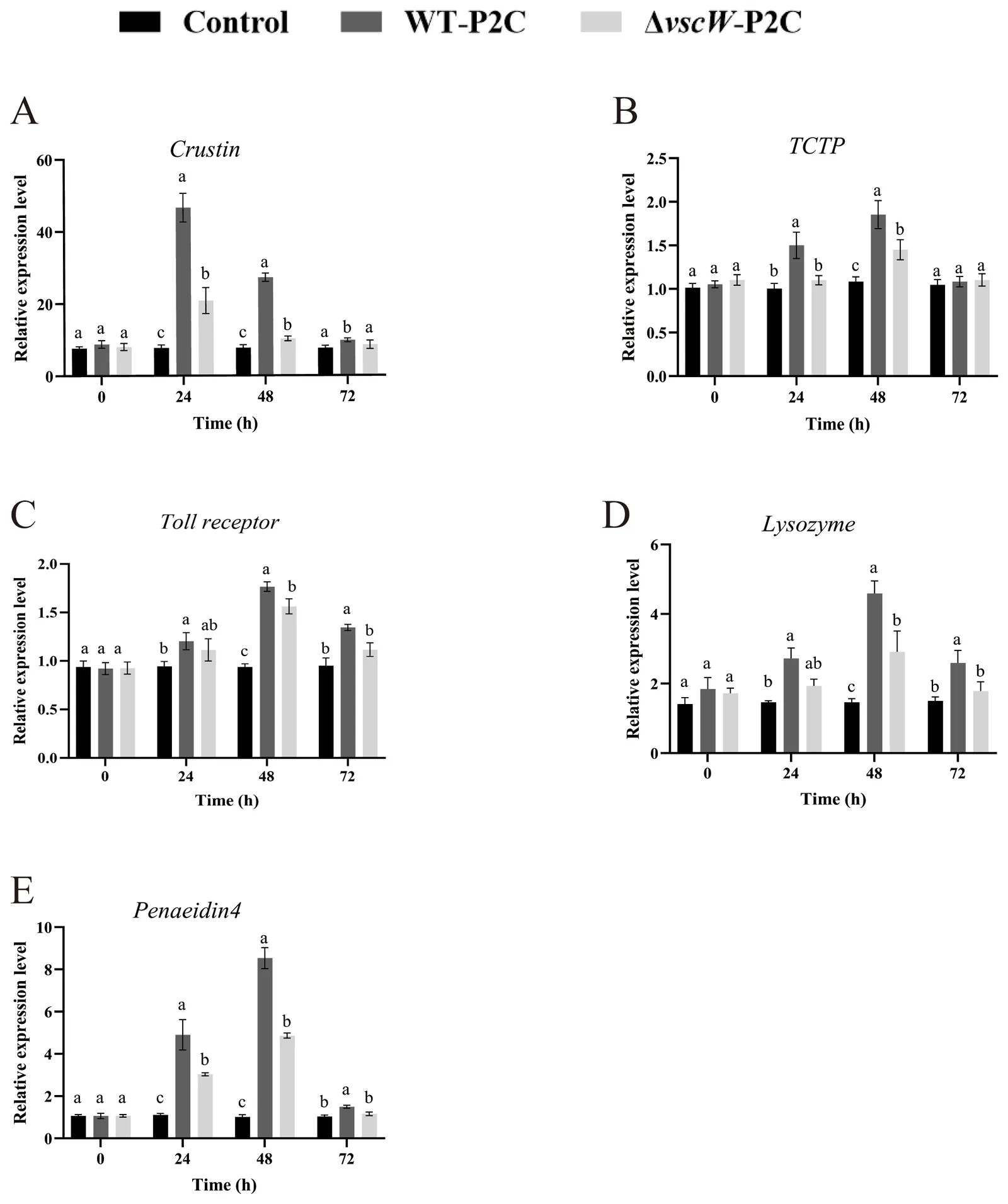
Effects of *vscW* deletion on immune-related gene expression in *Litopenaeus vannamei*. Relative expression levels of **(A)** *crustin*, **(B)** *TCTP*, **(C)** *Toll receptor*, **(D)** *lysozyme*, and **(E)** *penaeidin 4* in the hepatopancreas at different time points after bacterial challenge. Data are presented as the mean ± SD (n = 3 biological replicates per group at each time point), with each sample assayed in technical triplicate. Different lowercase letters **(a, b, and c)** at the same time point indicate significant differences among groups (P < 0.05), whereas identical letters indicate no significant difference.

The activities of all four immune-related enzymes (ACP, AKP, LZM, and SOD) markedly increased in the WT-P2C and *ΔvscW*-P2C groups (**Figure 9**). Activity peaked at 24 h and gradually decreased at 48 and 72 h after treatment. The increases were generally greater in the WT-P2C group, particularly at 24 h post-challenge. Although the *ΔvscW*-P2C group also showed increased hepatopancreatic immune enzyme activity, the response was weaker than that of the WT-P2C group. These results showed that WT-P2C infection markedly activated the nonspecific immune response in the hepatopancreas of Pacific white shrimp, whereas the immune enzyme response induced by *ΔvscW*-P2C was generally weaker than that induced by WT-P2C infection.

**Figure 9:**
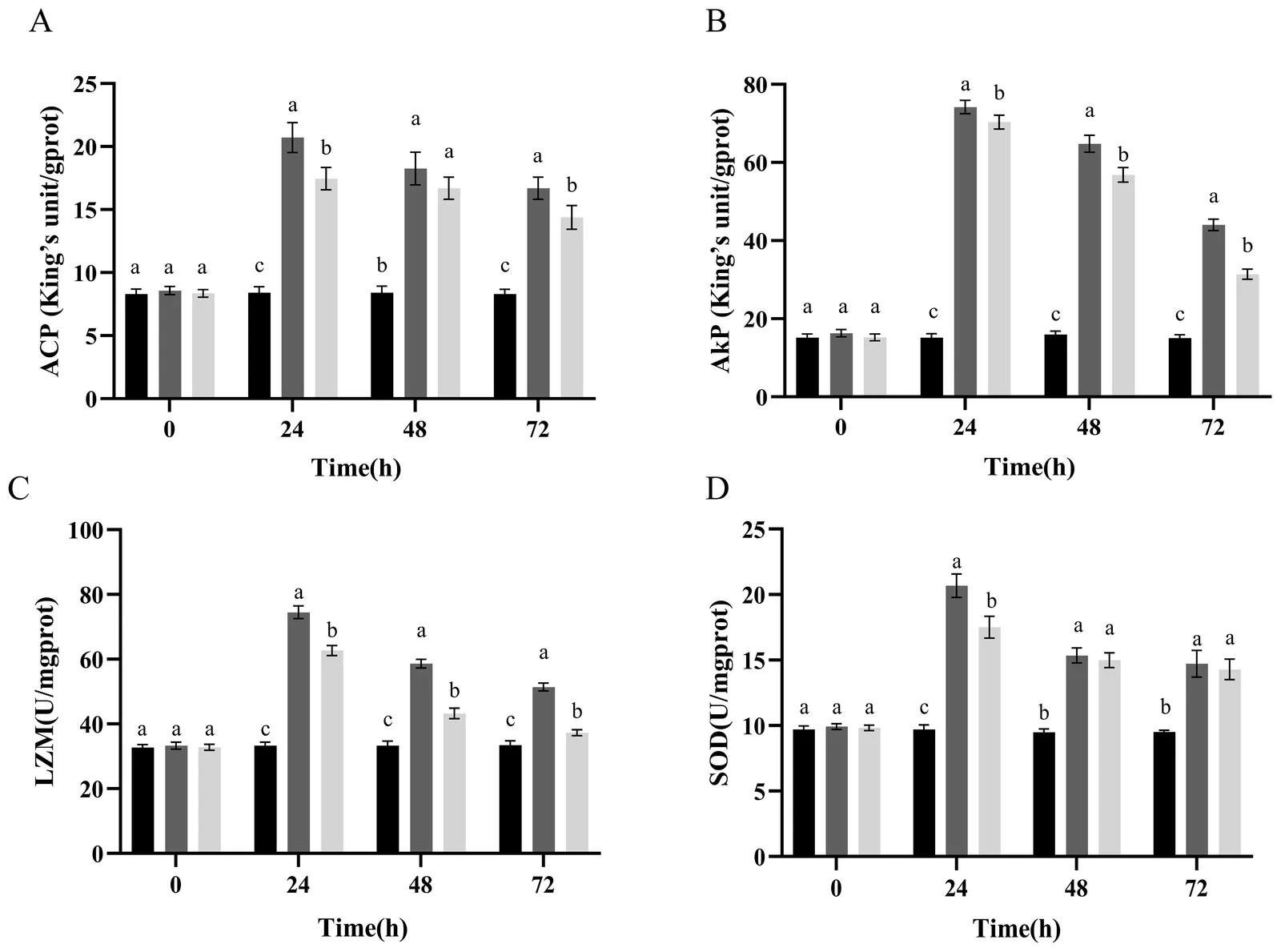
Effects of *vscW* deletion on immune-related enzyme activities in *Litopenaeus vannamei*. Activities of **(A)** acid phosphatase (ACP), **(B)** alkaline phosphatase (AKP), **(C)** lysozyme (LZM), and **(D)** superoxide dismutase (SOD) in the hepatopancreas at different time points after bacterial challenge. Data are presented as the mean ± SD (n = 3 biological replicates per group at each time point), with each sample assayed in technical triplicate. Different lowercase letters **(a, b, and c)** at the same time point indicate significant differences among groups (P < 0.05), whereas identical letters indicate no significant difference.

Collectively, these results show that *vscW* deletion reduced the pathogenicity of P2C in Pacific white shrimp.

## Discussion

*V. parahaemolyticus* is an important pathogen that is widely distributed in marine environments, and T3SS1 plays a major role in host infection and virulence. VscW is a T3SS1-associated accessory protein; however, its function in *V. parahaemolyticus* pathogenesis remains poorly understood. By constructing a deletion mutant (*ΔvscW*) and a complemented strain (C*ΔvscW*), we systematically evaluated the effects of *vscW* on the pathogenicity-associated phenotypes of strain P2C. Deletion of *vscW* markedly attenuated the pathogenicity of P2C toward shrimp and altered motility, biofilm formation, outer membrane homeostasis, and the expression of secretion system-related proteins. These results suggested that *vscW* contributes to virulence-associated phenotypes in *V. parahaemolyticus*, potentially through changes in T3SS1-related functions and membrane-associated physiological processes.

Bacterial motility is fundamental to environmental sensing, movement toward the host, and infection establishment. Flagella provide the principal structural basis for motility in *Vibrio* species and play important roles in successful bacterial colonization and survival within the host as well as in enabling bacteria to exert virulence (Haiko and Westerlund-Wikström, 2013). *V. parahaemolyticus* possesses two motility systems: polar and lateral. The polar flagellum primarily drives swimming in liquid environments and facilitates chemotaxis, nutrient acquisition, and host movement. In contrast, lateral flagella are closely associated with surface spreading and swarming motility and can promote bacterial dispersal, early adhesion, and colonization of host or environmental surfaces (Atsumi et al., 1992; McCarter, 2004; Echazarreta and Klose, 2019). Reduced motility affects bacterial colonization, survival, and virulence. Flagellar or motility-defective mutants of *V. vulnificus* exhibit reduced motility, impaired epithelial cell adhesion, defective biofilm formation, and decreased virulence in animal models (Lee et al., 2004). *Vibrio cholerae* possesses a single-sheathed polar flagellum that confers high motility. It establishes infection through motility and chemotaxis, and the direction of flagellar movement affects motility and, consequently, the efficiency of intestinal infection (Butler and Camilli, 2004). Thus, flagellum-mediated motility is closely associated with bacterial biofilm formation, colonization-associated phenotypes, and virulence (Khan et al., 2020). In this study, colony spreading by *ΔvscW*-P2C was reduced on medium containing 0.3% agar, whereas its swarming motility on medium containing 0.6% agar showed no evident change, suggesting that *vscW* deletion may exert a more pronounced effect on the swimming motility system. Transmission electron microscopy revealed fewer flagellar structures around *ΔvscW*-P2C cells, consistent with their reduced swimming motility. Therefore, we speculate that *vscW* deletion may affect flagellar formation, assembly, or stability, thereby influencing swimming motility and surface colonization of the host.

Biofilm formation facilitates bacterial adhesion to biotic or abiotic surfaces and enhances bacterial adaptation to nutrient limitations, antimicrobial agents, and host immune pressures (Hall-Stoodley et al., 2004; Sauer et al., 2022). In *Vibrio* species, biofilm formation is closely linked to virulence regulation. Flagellum-mediated motility facilitates bacterial movement toward and attachment to surfaces, after which extracellular matrix accumulation produces a relatively stable multicellular structure (Yildiz and Visick, 2009; Echazarreta and Klose, 2019; Zhong et al., 2022). In the present study, swimming motility and biofilm formation were concomitantly reduced in *ΔvscW*-P2C, suggesting an association between these two phenotypes. Following *vscW* complementation, biofilm formation was restored to a level approaching that of the wild-type strain, further supporting the involvement of *vscW* in this phenotype.

The outer membrane of gram-negative bacteria is an important barrier that maintains cell envelope homeostasis and restricts the entry of harmful hydrophobic molecules into cells (Delcour, 2009; Guest et al., 2021). NPN exhibited weak fluorescence in the intact outer membrane. When the barrier properties of the outer membrane were altered, more NPN entered the hydrophobic membrane, generating a stronger fluorescence signal. In this study, NPN fluorescence intensity increased in the *ΔvscW*-P2C strain, whereas the complemented strain recovered to a level approaching that of WT-P2C, indicating that *vscW* may contribute to the maintenance of the outer membrane barrier properties of P2C. VscW is homologous to *Yersinia* YscW, and YscW and related accessory proteins facilitate the localization and stabilization of T3SS secretin in the outer membrane. Therefore, *vscW* deletion may affect T3SS outer membrane components and indirectly influence cell envelope-associated processes.

Bacterial antimicrobial susceptibility is influenced by multiple factors, including drug entry, efflux pump activity, drug targets, inactivating enzymes, biofilms, metabolic state, and stress response regulatory networks (Zgurskaya and Rybenkov, 2020; Darby et al., 2023). For all 36 antimicrobial agents examined, *ΔvscW*-P2C and WT-P2C had the same susceptibility, intermediate, and resistance classifications, indicating that the deletion of *vscW* did not significantly alter the resistance profiles. Nevertheless, the larger inhibition zones observed for *ΔvscW*-P2C with several antibiotics suggest lower tolerance. When considered together with the increased outer membrane permeability detected by the NPN assay and the reduced biofilm formation by *ΔvscW*-P2C, these results suggest that *vscW* deletion may weaken the outer membrane barrier and collective protection, thereby facilitating the entry of antibiotics. However, these differences were insufficient to alter the bacterial resistance phenotypes.

VscW was predicted to be a T3SS1-associated accessory protein, and its homologue, YscW, promotes the stability and correct localization of the outer membrane secretin YscC. Therefore, *vscW* deletion may affect the assembly and secretion efficiency of T3SS. In the present study, multiple T3SS-related proteins were significantly altered in Δ*vscW*-P2C mutants. These proteins included the outer membrane protein VscC, the translocator-associated protein VopB, and proteins associated with T3SS needle assembly, which is consistent with our RT-qPCR results. Despite upregulation of several T3SS-related proteins in *ΔvscW*-P2C, the mutant showed reduced motility, biofilm formation, and *in vivo* virulence. Therefore, we suggest that the upregulation of these T3SS proteins may reflect a compensatory response to disrupted secretion system assembly or regulatory imbalance, rather than enhanced T3SS function.

Notably, *vscW* deletion altered not only T3SS-related proteins but also T6SS-related proteins and proteins involved in membrane transport, c-di-GMP signaling, and two-component signaling, with T6SS-related proteins showing particularly pronounced modular changes. T6SS is a contractile secretion system that resembles the tails of bacteriophages. It comprises an Hcp inner tube, VgrG/PAAR spike tip, TssB/TssC contractile sheath, TssE/F/G/K baseplate, and TssJ/L/M membrane complexes. This system can deliver effector proteins into neighboring bacteria or host cells, thereby contributing to interbacterial competition, environmental adaptation, competition within biofilms, and host interactions (Basler et al., 2012; Ho et al., 2014; Coulthurst, 2019). Deletion of *vscW* caused significant changes in multiple T6SS-related proteins in strain P2C, including the core structural and assembly proteins Hcp, VgrG, ClpV/VasG, ImpB, ImpC, VasD, VasL, ImpL/VasK, ImpJ, and DotU/VasF. Hcp is commonly considered a T6SS inner tube protein and a marker of secretory activity; VgrG is located at the T6SS spike tip and participates in effector delivery; ClpV/VasG functions as an ATPase involved in disassembling the contracted sheath and recycling the system; ImpB and ImpC form the contractile sheath; VasD, VasL, ImpL/VasK, and related proteins participate in transmembrane complex formation and apparatus anchoring (Silverman et al., 2013; Ho et al., 2014; Durand et al., 2015).

Several homologous T6SS proteins, including Hcp, VgrG, ClpV/VasG, and ImpC, showed opposing expression patterns at different loci. For example, one group of T6SS-related proteins, including *gene1416*/*hcp*, *gene1417*/*vgrG*, *gene1415*/*vasG*, and *gene1425*/*impC*, was significantly upregulated, whereas another group, including *gene4675*/*hcp*, *gene4674*/*vgrG*, *gene4676*/*clpV*, and *gene4682*/*impC*, was downregulated. This pattern may arise because many gram-negative bacteria carry multiple T6SSs. For example, *Pseudomonas aeruginosa* PAO1 carries three T6SSs: H1-T6SS, H2-T6SS, and H3-T6SS, whereas the T6SS of *Vibrio cholerae* can coexist with auxiliary effector modules (Unterweger et al., 2014; Allsopp et al., 2017). Thus, proteins with identical names or similar functions may be encoded at separate gene loci and belong to different T6SS gene clusters or submodules. Individual T6SS modules may contribute differently to bacterial competition, environmental adaptation, and host interactions and may be governed by distinct regulatory networks (Allsopp et al., 2017; Coulthurst, 2019). Accordingly, *vscW* deletion may differentially regulate T6SS subsystems and induce secretion system remodeling between T3SS and T6SS. T3SS is generally associated with host cell interactions and effector delivery, whereas T6SS participates in interbacterial competition, environmental adaptation, and specific host interactions. Therefore, changes in T6SS-related proteins may reflect an adaptive response to perturbation of secretion systems, although their biological significance requires further validation. A similar relationship has been reported in *P. aeruginosa*, in which the RetS sensory system can switch, through a regulatory network modulated by c-di-GMP signaling, between T3SS, which is associated with acute infection, and T6SS, which is associated with chronic infection and community lifestyles. These findings indicate that T3SS and T6SS do not function as isolated secretion apparatuses but are coordinated by shared regulatory networks (Goodman et al., 2004; Moscoso et al., 2011). Subsequent studies have shown that RsmA and AmrZ coordinate the assembly of all three T6SSs in *P. aeruginosa*, further indicating that T6SSs can be collectively regulated across different bacterial lifestyles and environmental stressors (Allsopp et al., 2017; Coulthurst, 2019). In this study, *vscW* deletion was accompanied by differential expression of multiple T6SS-related proteins; however, the underlying mechanism remains unclear.

The results of the Pacific white shrimp challenge provided *in vivo* evidence that *vscW* contributes to the pathogenicity of *V. parahaemolyticus*. Compared to the WT-P2C group, shrimp in the *ΔvscW*-P2C group had a higher final survival rate and showed relatively mild changes in body coloration and the appearance of the tail fan, intestine, and hepatopancreas. *V. parahaemolyticus* is an important opportunistic pathogen in shrimp aquaculture, and some virulent strains can cause hepatopancreatic injury, intestinal abnormalities, and high mortality. Virulence factors, secretion systems, motility, and colonization-associated phenotypes are closely associated with pathogenicity (Makino et al., 2003; Lee et al., 2015).

Regarding the host immune response, the expression of multiple immune-related genes, including lysozyme, Toll receptor, crustin, TCTP, and penaeidin 4, increased in the hepatopancreas of Pacific white shrimp infected with WT-P2C. These genes are involved in bacterial clearance, pattern recognition, signal transduction, antimicrobial peptide defense, and stress protection. Crustin and penaeidin are important antimicrobial peptide families in shrimp and are usually upregulated following *Vibrio* infection or bacterial stimulation to restrict pathogen proliferation. Lysozyme disrupts bacterial cell walls and participates in humoral immunity, whereas Toll-related pathways are involved in pathogen recognition and the induction of antimicrobial effector molecules (Jiravanichpaisal et al., 2006; Smith et al., 2008; Tassanakajon et al., 2013; Kulkarni et al., 2021). Compared to the WT-P2C group, the *ΔvscW*-P2C group showed an overall attenuation in the induction of these genes during the main immune response phase at 24–48 h post-challenge. ACP, AKP, LZM, and SOD activities increased following WT-P2C infection, indicating that pathogen stimulation can induce nonspecific immune and oxidative stress-related responses in shrimp. The increase in enzyme activity was generally weaker in the *ΔvscW*-P2C group, which was consistent with the lower immune gene responses and mortality observed in this group. These findings may be associated with lower pathogenicity or weaker host stimulation caused by the deletion mutant, which did not activate the host immune system as strongly as the wild-type strain. Together with the preceding results, these findings suggest that the attenuated virulence of *ΔvscW*-P2C may not result from a single factor, but from the combined weakening of multiple virulence-associated phenotypes.

In this study, we constructed a *vscW* deletion mutant and a complemented strain of *V. parahaemolyticus* P2C, and systematically evaluated the effects of *vscW* on bacterial physiological and pathogenicity phenotypes. Deletion of *vscW* did not markedly affect basal growth or the overall antimicrobial susceptibility of the strain; however, it decreased swimming motility and biofilm formation, increased outer membrane permeability, and was accompanied by changes in the expression of T3SS-related genes and proteins associated with secretion systems, membrane transport, motility, chemotaxis, and c-di-GMP signaling. Compared to WT-P2C, *ΔvscW*-P2C exhibited reduced lethality toward Pacific white shrimp and elicited a weaker host immune response. These results indicate that *vscW* participates in virulence-associated phenotypes and host infection by *V. parahaemolyticus* P2C, and that this role may be associated with coordinated changes in secretion system function, cell envelope homeostasis, motility, and biofilm formation. However, further investigations are required to confirm this hypothesis. This study provides a basis for further elucidating *vscW*-associated virulence regulatory mechanisms and offers insights into the regulation of bacterial virulence and suggest that VscW-associated functions may represent potential targets for anti-virulence intervention.

## Data Availability

The original contributions presented in the study are publicly available. The mass spectrometry proteomics data have been deposited to the ProteomeXchange Consortium via the iProX partner repository under accession number PXD083971 (iProX project ID: IPX0019554000).
